# A New Age-Structured Multiscale Model of the Hepatitis C Virus Life-Cycle During Infection and Therapy With Direct-Acting Antiviral Agents

**DOI:** 10.3389/fmicb.2018.00601

**Published:** 2018-04-04

**Authors:** Barbara de M. Quintela, Jessica M. Conway, James M. Hyman, Jeremie Guedj, Rodrigo W. dos Santos, Marcelo Lobosco, Alan S. Perelson

**Affiliations:** ^1^FISIOCOMP Laboratory, PPGMC, Universidade Federal de Juiz de Fora, Juiz de Fora, Brazil; ^2^Department of Mathematics and Center for Infectious Disease Dynamics, The Pennsylvania State University, State College, PA, United States; ^3^Mathematics Department, Tulane University, New Orleans, LA, United States; ^4^IAME, UMR 1137, Institut National de la Santé et de la Recherche Médicale, Université Paris Diderot, Sorbonne Paris Cité, Paris, France; ^5^Theoretical Biology and Biophysics, Los Alamos National Laboratory, Los Alamos, NM, United States

**Keywords:** computational biology, HCV, RNA, DAAs, differential equations

## Abstract

The dynamics of hepatitis C virus (HCV) RNA during translation and replication within infected cells were added to a previous age-structured multiscale mathematical model of HCV infection and treatment. The model allows the study of the dynamics of HCV RNA inside infected cells as well as the release of virus from infected cells and the dynamics of subsequent new cell infections. The model was used to fit *in vitro* data and estimate parameters characterizing HCV replication. This is the first model to our knowledge to consider both positive and negative strands of HCV RNA with an age-structured multiscale modeling approach. Using this model we also studied the effects of direct-acting antiviral agents (DAAs) in blocking HCV RNA intracellular replication and the release of new virions and fit the model to *in vivo* data obtained from HCV-infected subjects under therapy.

## Introduction

Chronic hepatitis C virus (HCV) infection affects about 130–150 million people worldwide and is the primary cause of liver cirrhosis and liver cancer (WHO, 2016). HCV has a linear positive strand RNA molecule with ~9,600 nucleotides as its genome and has been classified as belonging to the genus *Hepacivirus* in the *Flaviridae* family (Appel et al., 2006; Gastaminza et al., 2008). For many years HCV replication was not completely understood due to the inability to culture virus *in vitro*. However, the development of an HCV cell culture (HCVcc) system has allowed investigation of the processes that govern HCV replication and other features of its life cycle (Appel et al., 2006; Elliot et al., 2009; Afzal et al., 2015). Moreover, new means of distinguishing and quantifying both positive and negative HCV RNA strands have been developed and improved (Bessaud et al., 2001; Craggs et al., 2001).

HCV primarily infects liver cells, called hepatocytes. After entry into a hepatocyte, the positive strand HCV RNA is uncoated and translated into a polyprotein from which all HCV proteins are produced. The HCV NS5B RNA-dependent RNA polymerase copies the positive HCV RNA into one or more HCV RNA negative strands. The nonstructural HCV proteins together with negative strand HCV RNA form replication complexes, the molecular machines responsible for producing more positive strands of HCV RNA (Quinkert et al., 2005). The newly produced positive strands can either be used for translation, replication or be assembled into virus particles and exported from the infected cell. How the decision among the options is made remains unclear (Appel et al., 2006; Elliot et al., 2009; Bisceglie, 2010). HCV RNA replication depends not only on HCV proteins but host factors also play an important role (Scheller et al., 2009; Jangra et al., 2010).

Guedj et al. (2013) developed an age-structured multiscale model of HCV infection and treatment including the dynamics of intracellular viral RNA (vRNA). The model has been analyzed mathematically and various approximate solutions derived (Rong et al., 2013; Rong and Perelson, 2013).

Age-structured models have been widely used to study the epidemiology of infectious diseases, such as HIV (Thieme and Castillo-Chavez, 1993), hepatitis C (Martcheva and Castillo-Chavez, 2003) and tuberculosis (Castillo-Chavez and Feng, 1997; Thieme and Castillo-Chavez, 2002). Nelson et al. (2004) presented an age-structured model of the dynamics of within host HIV. Gilchrist et al. (2004) used an age-structured model to explore how the intracellular HIV production rate influenced the virus' fitness. One advantage of using such an approach is the possibility of considering that individuals or cells with distinct ages could behave differently (Li and Brauer, 2008). Using that approach in modeling the dynamics of virus within a host allows a realistic representation of infection biology in which the rate of production and release of new virus is not constant but rather depends on the length of time a cell has been infected. Moreover, the model can also account for an infected cell death rate that depends on the time the cell has been infected.

The Guedj et al. (2013) age-structured multiscale model of HCV infection only considered the dynamics of positive strand HCV RNA. Guedj and Neumann (2010) studied the intracellular dynamics of both positive- and negative-strand viral RNA. They used ordinary differential equations to represent the number of positive-strands of viral RNA, available for transcription and translation, and the number of negative-strands of viral RNA or “replication units.” Benzine et al. (2017) developed a more detailed ordinary differential equation model in which they distinguished positive strand HCV RNA used for translation, replication and viral assembly. However, both Guedj and Neumann (2010) and Benzine et al. (2017) did not consider that the number of positive and negative strands of viral RNA depend on how long a cell has been infected.

Here we used a three-equation age-structured model for intracellular HCV RNA dynamics, introduced by Quintela et al. (2017), which incorporated negative strand HCV RNA as well as the positive-strand HCV RNA available for translation and replication separately and validated the model by comparison to *in vitro* experiments. We then coupled this intracellular model to a well-established cell infection model and showed the model was able to fit *in vivo* viral load data obtained from patients treated with direct acting antiviral (DAA) therapy.

## Materials and methods

### Intracellular model of HCV replication

**Figure 1:**
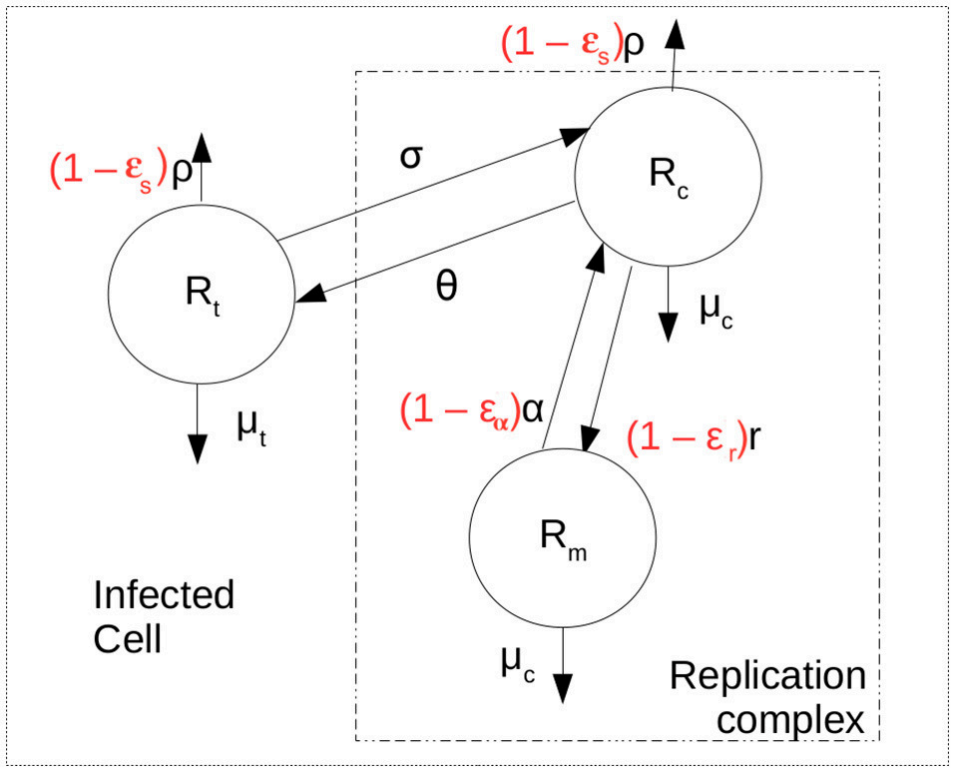
Intracellular model scheme. After cell entry positive strand HCV RNA is available for translation, represented by *R*_*t*_. It can be exported at rate ρ and decay at rate μ_*t*_. Negative or minus strand HCV RNA (*R*_*m*_) is produced at maximum rate *r* and forms the replication complexes that produce more positive strand RNA (*R*_*c*_) at rate α. It is assumed that HCV RNA inside the replication complex in both orientations have the same decay rate μ_*c*_. The positive strand HCV RNA available for translation is assumed to move into replication complexes at rate σ and from replication complexes at rate θ. The terms in red represent the action of therapy in blocking secretion and production of viral RNA.

We developed a mathematical model to represent the intracellular replication of HCV shown schematically in Figure 1. The model allows the study of aspects such as translation of positive-strand HCV RNA after cell entry, transfer of the positive strand to the membranous web where it is used for replication, production of negative- and positive-strand HCV RNA within replication complexes and secretion of positive-strand RNA as virions. The replication of HCV RNA has been studied in detail c.f. (Chatel-Chaix and Bartenschlager, 2014; Li et al., 2015).

The system of ordinary differential equations used to represent the dynamics of intracellular infection over time is

(1) $$\begin{array}{l} \left\{ \begin{array}{c} \frac{d}{da}R_{t}=\theta R_{c}-\left(\sigma +\rho \left(a\right)+\mu_{t}\right)R_{t},\;\\ \frac{d}{da}R_{c}=\alpha R_{m}+\sigma R_{t}-\left(\theta +\rho \left(a\right)+\mu_{c}\right)R_{c},\;\\ \frac{d}{da}R_{m}=r\left(1-\frac{R_{m}}{R_{max}}\right)R_{c}-\mu_{c}R_{m},\; \end{array}\right. \end{array}$$

$$\begin{array}{l} R_{t}\left(0\right)=R_{t_{0}},R_{c}\left(0\right)=0,R_{m}\left(0\right)=0, \end{array}$$

where *R*_*t*_ represents positive strand HCV RNA used for translation, *R*_*c*_ represents positive strands within replication complexes used for replication, *R*_*m*_ represents minus (or negative) strand RNA and *a* represents the time a cell has been infected. Positive strand HCV RNA forms the viral genome. After cell entry, cellular machinery translates this positive strand RNA into a polyprotein in the cytoplasm (Shi and Lai, 2006). However, after polyproteins are made the positive strand must also be used for replication and must be copied into minus stand RNA. We assume that the positive-strand HCV RNA used for translation (*R*_*t*_ in Equation 1) moves from the cytoplasm into what is called the membranous web and interacts with the proteins needed for replication to become a species we call *R*_*c*_ at rate σ per strand. We also assume the positive strand in the cytoplasm, *R*_*t*_ has a natural decay rate of μ_*t*_ per strand. Lastly, positive strands need to be assembled into virions which are then exported from the infected cell. Virion assembly occurs in association with cytosolic lipid droplets (Chatel-Chaix and Bartenschlager, 2014). As it is not clear whether the positive strand RNA in the membranous web needs first to be transported into the cytosol for viral assembly, we assume both *R*_*t*_ and *R*_*c*_ can be assembled into virions and exported at rate ρ(*a*). The time-dependence of ρ will be discussed below. Further, we assume positive-strand HCV RNA in the replication complex (*R*_*c*_) can move out of the replication complex and membranous web and back into the cytoplasm to become *R*_*t*_ at rate θ. More detailed models can be developed that separate virion assembly from secretion and that include a separate compartment of positive strand RNA used for virion assembly (cf. Benzine et al., 2017), but here for simplicity we have combined these steps.

Minus-strand HCV RNA (*R*_*m*_) is formed in the replication complex by copying the positive strand *R*_*c*_ at maximum rate *r*. As in Guedj and Neumann (2010), it is assumed that host factors limit the replication of negative-strand RNAs, so that as the maximum number R_*max*_ is reached replication slows according to a logistic growth law. The positive strands in replication complexes, *R*_*c*_, are copied from the negative strand template at rate α per template. We consider that both *R*_*c*_ and *R*_*m*_ are in the replication complex and decay at the same *per capita* rate μ_*c*_.

In order to have a positive equilibrium when the model represents an established infection, the parameters need to satisfy the relations: $\phi_{2}>\frac{\sigma \theta }{\phi_{1}}$ and $\alpha r>\left(\phi_{2}-\frac{\sigma \theta }{\phi_{1}}\right)\mu_{c}$ in which ϕ_1_ = θ + ρ + μ_*t*_ and ϕ_2_ = σ + ρ + μ_*c*_.

#### Delay in particle assembly

Following translation and replication, positive-strand HCV RNA is assembled into a virus particle that can then be exported out of the cell (Lindenbach and Rice, 2013). Such assembly can not begin immediately after infection as viral proteins are needed as components of the virion and hence first need to be produced. The release of virus by an infected cell *in vitro* is observed approximately 12 h after infection (Keum et al., 2012).

To incorporate this biological delay, τ, we assume the viral secretion rate is a function of the length of time a cell has been infected, i.e., its age of infection. The function we use is

(2) $$\begin{array}{l} \rho \left(a\right)=\left\{ \begin{array}{c} 0,a<\tau \;\\ \left(1-e^{-k\left(a-\tau \right)}\right)\rho ,\text{otherwise},\; \end{array}\right. \end{array}$$

where *a* = 0 is the time of infection and the constant ρ is the maximum secretion rate. This functional form was chosen to avoid any discontinuities.

When we analyze *in vitro* experiments, the kinetics of secreted HCV RNA, *R*_*s*_ can be represented by the differential equation

(3) $$\begin{array}{l} \left\{ \begin{array}{c} \frac{d}{da}R_{s}=\rho \left(a\right)\left(R_{t}+R_{c}\right)-c_{s}R_{s}\;\\ R_{s}\left(0\right)=0,\; \end{array}\right. \end{array}$$

where ρ(*a*) is the secretion rate and *c*_*s*_ is the rate of clearance or degradation of secreted HCV RNA, which is estimated from the data.

### Coupling of multiple scales

**Figure 2:**
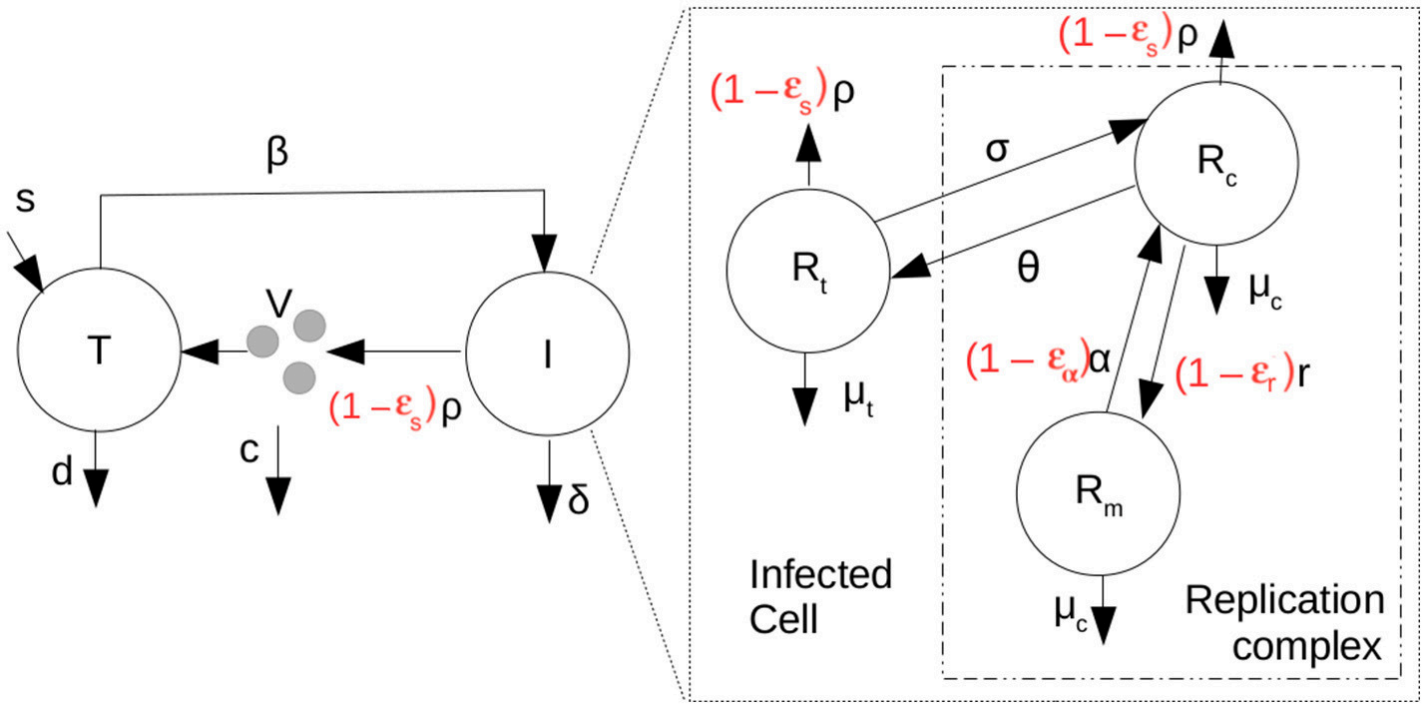
Scheme representing the coupled multiscale model with therapy (parameters in red). T are target cells, I, infected cells and V, the HCV RNA concentration in plasma. Target cells become infected at rate β.

We also analyze *in vivo* data in which the effects of antiviral treatment on kinetics of HCV RNA levels in plasma are measured. To fit this data we introduced a new multiscale model depicted in Figure 2.

The intracellular portion of the multiscale model with treatment is represented by the following partial differential equations (PDEs) in which *t* represents clock time and *a* the age of an infected cell:

(4) $$\begin{array}{l} \left\{ \begin{array}{c} \frac{\partial }{\partial t}R_{t}\left(a,t\right)+\frac{\partial }{\partial a}R_{t}\left(a,t\right)=\theta R_{c}-\left(\sigma +\left(1-\epsilon_{s}\right)\rho \left(a\right)+\kappa_{t}\mu_{t}\right)R_{t},\;\\ \frac{\partial }{\partial t}R_{c}\left(a,t\right)+\frac{\partial }{\partial a}R_{c}\left(a,t\right)=\left(1-\epsilon_{\alpha }\right)\alpha R_{m}+\sigma R_{t}-\;\\ \left(\theta +\left(1-\epsilon_{s}\right)\rho \left(a\right)+\kappa_{c}\mu_{c}\right)R_{c},\;\\ \frac{\partial }{\partial t}R_{m}\left(a,t\right)+\frac{\partial }{\partial a}R_{m}\left(a,t\right)=\left(1-\epsilon_{r}\right)r\left(1-\frac{R_{m}}{R_{max}}\right)R_{c}-\kappa_{c}\mu_{c}R_{m},\; \end{array}\right.\\ R_{t}\left(0,t\right)=R_{t_{0}},R_{t}\left(a,0\right)={\bar{R}}_{t}\left(a\right),\\ R_{c}\left(0,t\right)=0,R_{c}\left(a,0\right)={\bar{R}}_{c}\left(a\right),\\ R_{m}\left(0,t\right)=0,R_{m}\left(a,0\right)={\bar{R}}_{m}\left(a\right). \end{array}$$

We have assumed that intracellular infection is initiated by the introduction of *R*_*t*_0__ positive HCV RNA strands into the cytoplasm of a cell. Typically, we shall assume that infection is the result of a single virion, carrying a single positive-strand HCV RNA, entering a cell, so that *R*_*t*_0__ = 1. Further, we shall assume that the individual's being treated with antivirals are chronically infected and have reached steady state in which ${\bar{R}}_{t}\left(a\right)$, ${\bar{R}}_{c}\left(a\right)$ and ${\bar{R}}_{m}\left(a\right)$ are the steady state distributions of positive-strand HCV RNA in translation and in replication complexes and negative-strand HCV RNA in replication complexes, respectively, in the absence of treatment and are given by the steady state solutions of the ODEs in Equation (1). Further, we let ϵ_α_ be the effectiveness of therapy in decreasing or blocking positive-strand RNA replication, ϵ_*r*_ the effectiveness of therapy in decreasing or blocking negative-strand RNA replication, and ϵ_*s*_ the effectiveness of therapy in decreasing or blocking secretion of positive-strand RNA, where for each of the ϵ's, ϵ = 1 corresponds to a 100% effective drug and ϵ = 0 corresponds to a completely ineffective or absent drug. Further κ_*t*_ is a factor by which therapy changes the degradation rate of positive-strand RNA used for translation and κ_*c*_ is the factor by which therapy changes the degradation rate of both positive and negative strand RNA in replication complexes.

To complete the multiscale model, we then coupled the intracellular model to an established HCV cellular infection model (Equation 5) (Neumann et al., 1998; Canini and Perelson, 2014).

(5) $$\begin{array}{l} \left\{ \begin{array}{c} \frac{d}{dt}T\left(t\right)=s-\beta V\left(t\right)T\left(t\right)-dT\left(t\right),\;\\ \frac{\partial }{\partial t}I\left(a,t\right)+\frac{\partial }{\partial a}I\left(a,t\right)=-\delta \left(a\right)I\left(a,t\right),\;\\ \frac{d}{dt}V\left(t\right)=\left(1-\epsilon_{s}\right)\overset{\infty }{\underset{0}{\int }}\rho \left(a\right)\left(R_{t}\left(a,t\right)+R_{c}\left(a,t\right)\right)I\left(a,t\right)da-cV\left(t\right),\; \end{array}\right.\\ T\left(0\right)=T_{0},\\ I\left(0,t\right)=\beta V\left(t\right)T\left(t\right),I\left(a,0\right)=Ī\left(a\right),\\ V\left(0\right)=V_{0}, \end{array}$$

in which, *T* are target cells, *I*, infected cells and *V* the HCV RNA concentration in plasma. Target cells become infected at rate β, have a constant source rate *s* and a natural per capita decay rate *d*. The parameter δ(*a*) represents the death rate of an infected cell of age “*a*” and the effects of therapy on the virus export are given by ϵ_*s*_. Here for simplicity we shall only analyze the case in which δ(*a*) is a constant, δ. Virus in the plasma is cleared from the circulation at per capita rate *c*. Here we have assumed that at *t* = 0, the time therapy starts, the system is in steady state, where Ī(*a*) is the steady-state distribution of infected cells, which can be shown to be *Ī*(*a*) = $\beta V_{0}T_{0}e^{-\delta a}$. $T_{0}=\frac{c}{\beta N}$, where *N* is the steady state total amount of virus secreted by an infected cell over its lifetime, $N=\rho \int_{0}^{\infty }({\bar{R}}_{t}(a)+{\bar{R}}_{c}(a)e^{-\delta a}da$, and $V_{0}=\frac{s-dT_{0}}{\beta T_{0}}$. See Rong et al. (2013). The coupling between the intracellular and extracellular models occurs through the equation for *V*, the virus in the plasma. The amount of plasma virus depends on the number of infected cells and number of virions being packaged and exported per infected cell. This coupling has been used before (Guedj et al., 2013; Rong et al., 2013; Rong and Perelson, 2013).

### Numerical algorithms

The model equations were discretized in space, i.e., age, and integrated in time using the method of lines (MOL) approach (Sadiku and Obiozor, 2000; Shakeri and Dehghan, 2008) where the partial derivatives in age were approximated by finite-differences and the solution at the grid points was integrated along lines in time. We integrated the equations using the Matlab® ordinary differential equation Runge-Kutta solver *ode*45.

The domain was discretized on a uniform grid of 201 mesh points between ages 0 and 50 days, as it is unlikely for an infected cell to live longer than this. The boundary of domain at *a* = 50 was defined as a simple outflow boundary condition and was incorporated into the numerical solution by linearly extrapolating the solution to two buffer grid points outside the domain. The partial derivatives in age were approximated with fourth-order centered finite differences.

We verified the convergence of the numerical solution to an accuracy of 10^−3^ by varying the number of spatial grid points and the time integration error tolerance.

The simulation time was varied according to the length of time that virus was detected in plasma after therapy initiation in the data we analyzed. The computer run time were typically a few seconds for a single simulation using a laptop computer.

We used the Matlab® nonlinear optimization program *fmincon* to fit the solutions of the model to the experimental data by minimizing the *L*_2_ norm of the residual difference between the model solution and the data. This routine was chosen due to the possibility of specifying lower and upper bounds for the parameters we wanted to estimate. The algorithm we used was “*interior-point”* as it satisfies the bounds at all iterations.

The data we fit to validate the model was obtained from different sources. We extracted *in vitro* data from Keum et al. (2012) and Binder et al. (2013) using the on-line tool WebPlotDigitizer (Rohatgi, 2016). We also fit clinical trial data from Guedj et al. (2013) that we had access to.

Because our models have a large number of parameters we numerically approximated the Hessian of the objective function at the optimal parameter values. At a minimum, the gradient of the objective function is zero. If an eigenvalue of the Hessian is zero at the minimum, then the gradient remains zero along the direction of the associated eigenvector. That is, the solution is not unique (identifiable) (Beck, 2014). Here, for each of the data sets we fit, at the optimum, all of the eigenvalues of the Hessian were positive, and the condition number was below 10^4^, indicating that the parameters were locally identifiable.

## Results

### Calibrating intracellular parameters in the absence of therapy

To validate the intracellular mathematical model, we first compared the results of Equation (1) to transfection experiments performed by Binder et al. (2013). In that paper the authors used two distinct cell lines to assess HCV RNA replication over 72 h: (a) Huh7-Lunet cells which are highly permissive to HCV RNA replication and (b) Huh7 cells (Huh7-lp) which presents lower levels of HCV RNA replication. They measured positive-strand and negative-strand RNA by strand specific quantitative Northern blotting. Binder et al. (2013) developed a complex mathematical model that included 13 molecular species with 16 parameters in two compartments: the cytoplasm and a replication compartment.

**Figure 3:**
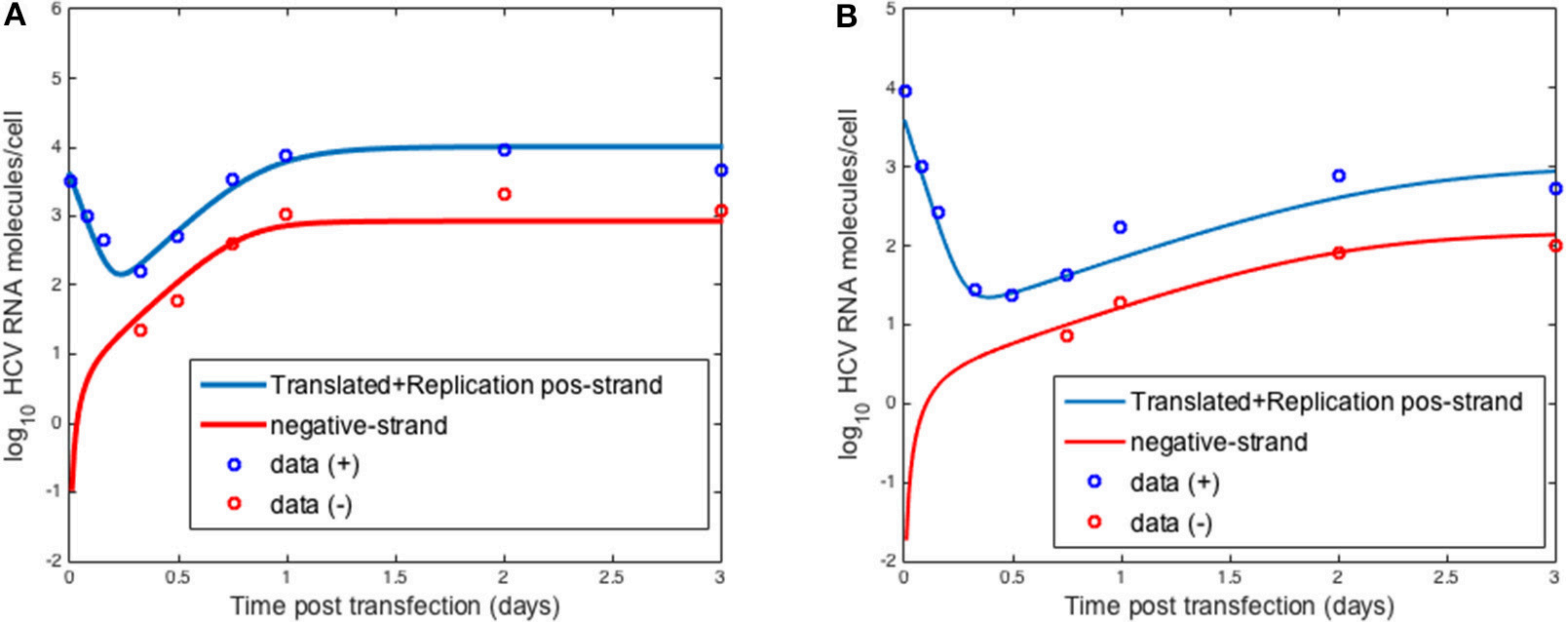
HCV RNA replication. Circles represent experimental data from Binder et al. (2013) and lines show the results obtained with the model described herein with distinct sets of parameters for **(A)** high and **(B)** low permissive cells.

Using the three equation mathematical model, Equation (1), we were able to fit the dynamics of both positive and negative strand HCV RNA in both the high and low permissive cell lines (Figure 3). Our model was able to replicate the initial decay seen after transfection with both types of cells and the plateau during the 72 h measured (Figures 3A,B).

In fitting the data, the parameters used to describe the age-dependent virion export rate, ρ(*a*), were fixed at ρ = 0.1 d^−1^, τ = 0.5 d^−1^ and *k* = 0.8 d^−1^. We set τ at 0.5 d^−1^ based on the fact that Keum et al. (2012) could not detect any extracellular virus until 12 h post-infection. We further tested different values of τ and *k* and chose the values that gave the best fits to the data in both the Binder and Keum experiments. Choosing the export rate as a time-dependent function rather than a constant allowed us to have an initial delay followed by a smooth transition to the maximum export ρ. Regarding the maximum export rate, ρ, we at first chose the value estimated by Guedj et al. (2013) based on fitting *in vivo* data. However, using this value did not give good fits to the *in vitro* data. We then scanned through different values and chose the one giving the best fit. HCV uses the host cell export machinery and thus it is not surprising that these parameters differ between *in vitro* and *in vivo* systems.

The initial number of HCV positive strands introduced into these cells to initiate HCV replication in this *in vitro* system was *R*_*t*_0__ = 4, 000 molecules cell^−1^. Other parameters of the model were estimated using the *fmincon* routine in Matlab and are shown in Table 1.

**Table 1:** Model parameter values estimated for the *in vitro* transfection experiments in Binder et al. (2013).

**Name**	**Huh7-Lunet**	**Huh7-lp**	**Unit**	**Biological meaning**
α	60	20	Day^−1^	R_*c*_ replication rate
μ_*t*_	20	20	Day^−1^	R_*t*_ natural decay rate
*r*	2.1	1	Day^−1^	R_*m*_ replication rate
μ_*c*_	3.4	1.7	Day^−1^	Repl. complex decay rate
σ	0.3	0.1	Day^−1^	Translation to repl. rate
θ	2.1	1.2	Day^−1^	Repl. to translation rate
*R*_*max*_	1000	200	Molecules cell^−1^	Max. number of R_*m*_

**Figure 4:**
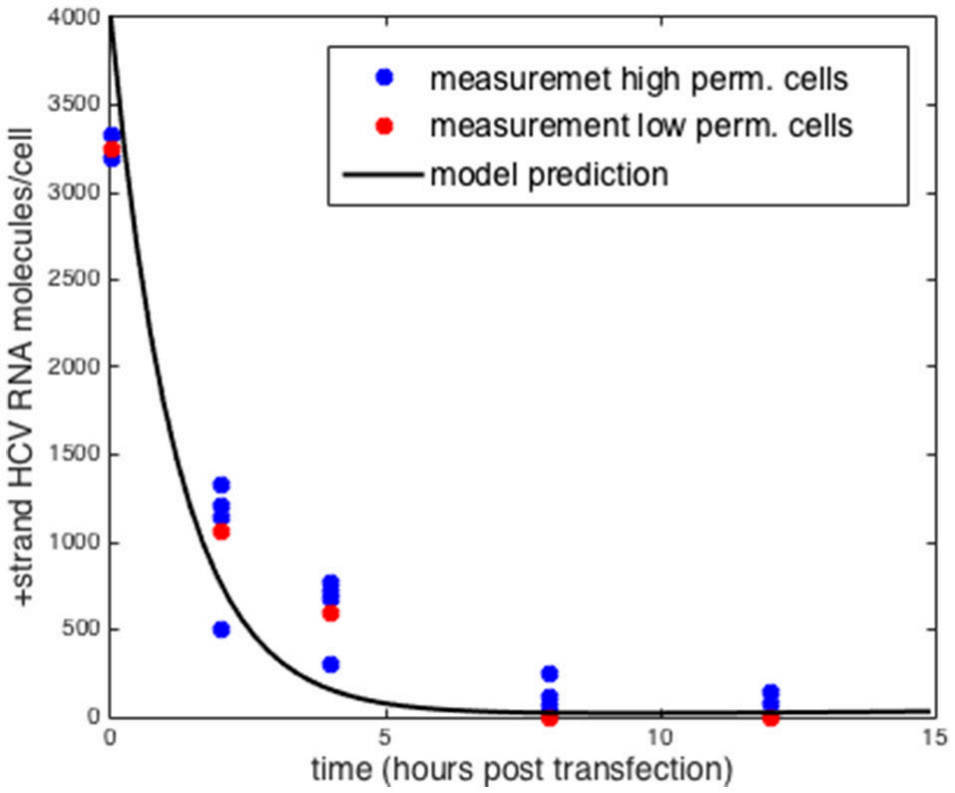
Comparison to measurements of replication deficient HCV RNA in high and low permissive cells. Model prediction setting σ = 0 for both sets of parameters. Data taken from Binder et al. (2013).

Another form of validation we performed was testing the model predictions by comparing to positive-strand measurements using a replication deficient replicon (Binder et al., 2013). By setting the rate at which positive-strand RNA goes from use in translation to use in replication (σ) to zero we could compare the results obtained with the model to the measurements reported by Binder et al. (2013) Without replication, the initial amount of transfected HCV RNA decays exponentially and no negative-strand is formed. Further as Binder et al. show the decay of positive strand RNA is similar in both the high permissive and low permissive cell lines. We simulated the intracellular model with the parameters that were estimated for the highly-permissive cell line (Table 1) and the results are shown in Figure 4. The results using the parameters for the low permissive cell line are the same.

#### Sensitivity analysis of the intracellular model

Forward sensitivity analysis was performed to estimate how the model solution is affected by small perturbations to each model parameter. The sensitivity index was defined as the ratio:

(6) $$\begin{array}{l} S_{i}=\frac{\delta J/J}{\delta p/p},J,p\neq 0 \end{array}$$

in which, *J* denotes a model output that depends on a parameter *p*, δ is some perturbation to the parameter *p* and δ*J* is the resulting perturbation to the output *J*.

**Figure 5:**
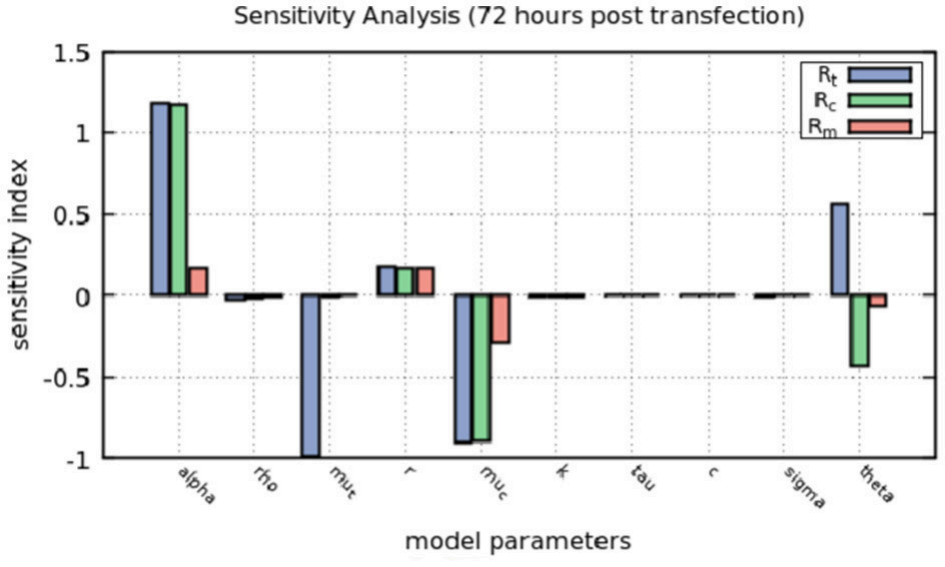
Sensitivity analysis of the model at 72 h. The positive-strand RNA replication rate, α, the natural decay rates for positive-strand RNA used for translation and within replication complexes, μ_*t*_ and μ_*c*_, repectively and the rate at which positive-strand RNA goes from replication complexes to the cytoplasm to be translated, θ, are the most sensitive parameters in the model.

The sensitivity index is a measure of the percentage of change in the output given a perturbation in each parameter. We varied by 10% the value of each parameter, while other parameters were kept the same, and calculated the sensitivity index of each parameter to the resulting value of *R*_*t*_, *R*_*c*_ and *R*_*m*_ at 72 h (Figure 5). Positive values indicate an increase in the output given the increase in the parameter and negative values indicate that the output decreases as we increase the parameter.

The sensitivity index confirms that perturbing α, the positive-strand RNA replication rate, increases by more than 10% the amount of positive-strand RNA used for translation and in replication complexes. μ_*t*_ represents the natural decay rate of translated RNA and changes in that parameter decreases positive-strand RNA in translation and μ_*c*_, the natural decay rate for both positive and negative strands in the replication complex, affects mainly the positive-strand RNA.

### Calibrating the intracellular parameters for a different *in vitro* experiment

**Figure 6:**
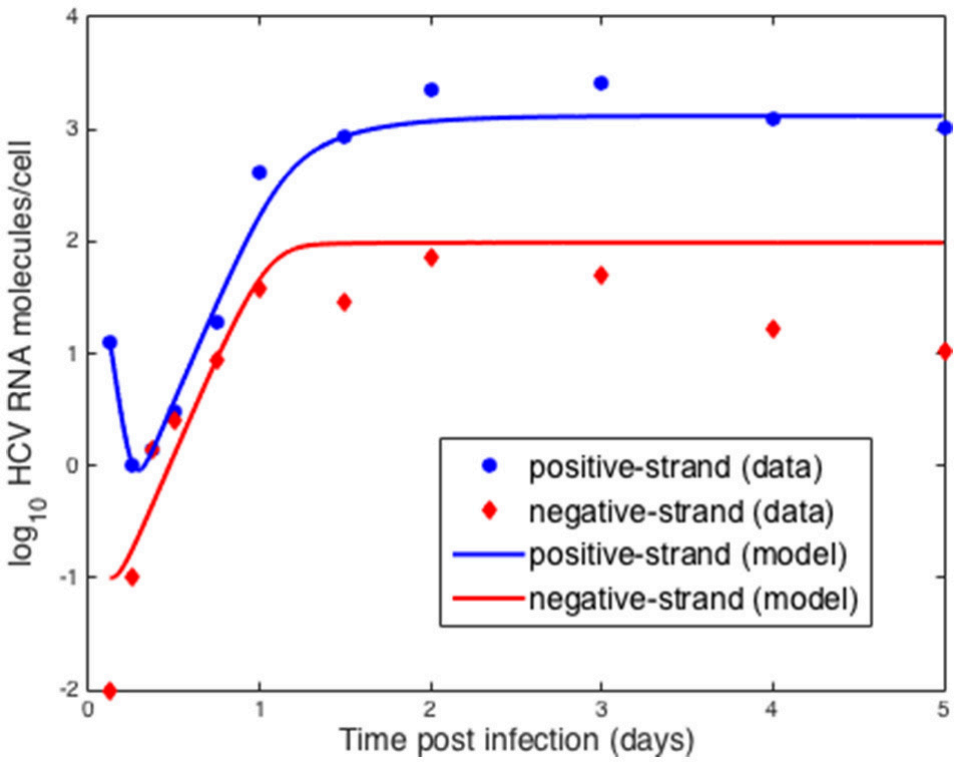
Comparison of model results to *in vitro* infection data. Data points were extracted from Keum et al. (2012) and the lines were obtained by fitting the intracellular model to the data where we assumed the measured positive strands were the sum of the positive strands used for translation, *R*_*t*_ and in replication complexes, *R*_*c*_. As before we fixed the export rate with ρ = 0.1 d^−1^, τ = 0.5 d^−1^, and *k* = 0.8 d^−1^. The initial time *t*_0_ = 0. Based on the data we set R_*t*_0__ = 12.8. Other parameters were estimated and were found to be α = 30 d^−1^, μ_*t*_ = 24 d^−1^, *r* = 3.18 d^−1^, μ_*c*_ = 1.05 d^−1^, *R*_*max*_ = 100 molecules, σ = 0.1 d^−1^ and θ = 1.2 d^−1^.

**Figure 7:**
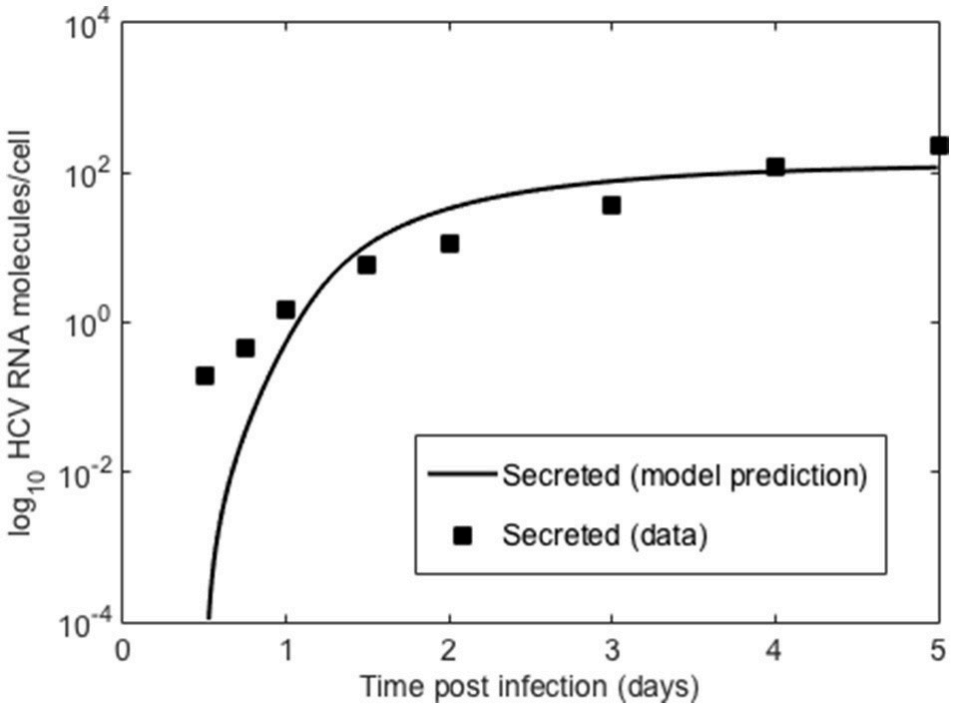
Secreted HCV RNA. Data points from Keum et al. (2012) and lines are the model prediction based on Equation (1).

We also compared the intracellular model to experiments *in vitro* performed by Keum et al. (2012) in which a high multiplicity of infection was used (MOI = 5 or 6) so that only one round of infection occurred. Theoretically, with an MOI of 5, 99.3% of cells should be infected with a least one infectious virion (Keum et al., 2012). A cell culture adapted HCV, JFH-m4, was incubated with Huh7.5.1 cells for 3 h to initiate infection. At subsequent times cells and supernatant were harvested to measure HCV RNA levels intra-cellularly and the amount secreted into the medium. Keum et al. quantified the number of positive and negative HCV RNA strands using real-time RT-PCR. As shown in Figure 6 the number of cell-associated positive strands initially decreased reaching a minimum of about 1 positive strand per cell at 6 h post-infection (pi). Intracellular negative strand, which serves as a template for making new positive strands, was first detected at 6 h pi. Our model was able to reproduce the observed intracellular HCV RNA dynamics (Figure 6) as well as the dynamics of positive strand HCV RNA secreted into the media (Figure 7). As before we fixed the export rate with ρ = 0.1 d^−1^, τ = 0.5 d^−1^, and *k* = 0.8 d^−1^. The initial time *t*_0_ = 0, R_*t*_0__ = 12.8 and no therapy was given (Figure 7). Other parameters were estimated and were found to be α = 30 d^−1^, μ_*t*_ = 24 d^−1^, *r* = 3.18 d^−1^, μ_*c*_ = 1.05 d^−1^, *R*_*max*_ = 100 molecules, σ = 0.1 d^−1^ and θ = 1.2 d^−1^. As both the cell line and virus used in these experiments are different than the ones used by Binder et al. (2013), it is surprising that resulting parameters do not differ very much from those we estimated in the previous section for high and low-permissive cells.

### *In vivo* effect of therapy with an NS5A inhibitor

We validated the coupled multiscale model by fitting Equations (4) and (5) to data obtained from patients treated with one dose of 10 or 100 mg of daclatasvir (DCV) (Guedj et al., 2013). DCV inhibits the action of the HCV NS5A protein, which has been shown to play an important role in HCV RNA replication and secretion (Lee, 2013; Scheel and Rice, 2013). This data was previously analyzed by Guedj et al. (2013) using a much simpler multiscale model that only considered HCV positive strand RNA dynamics.

We assumed that the parameters that represent *in vivo* infection dynamics are different from those we estimated for *in vitro* infection as both the virus and target cells are different. We also assumed that there was no superinfection, so that only one virus infects each cell. Using the same approach as for the intracellular model, we performed a sensitivity analysis of the coupled model parameters in order to determine how sensitive the predicted viral load is to each parameter. We chose to vary each parameter one at a time and compared how they affected the predicted viral load at day 2 on therapy.

**Figure 8:**
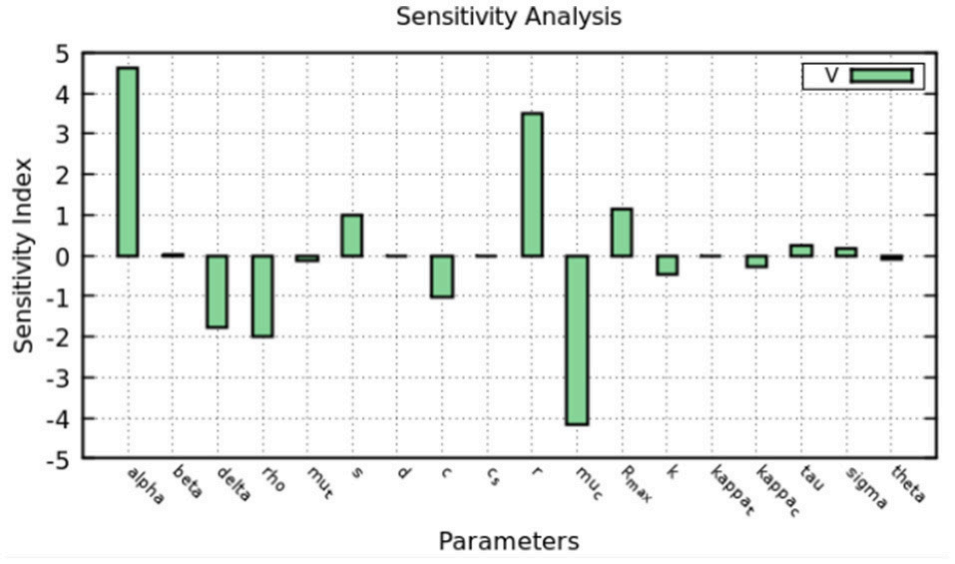
Sensitivity analysis of the model at 2 days. The figure shows how much a perturbation of the parameters influence the viral load (V).

The sensitivity index was calculated using Equation (6) and the results are shown in Figure 8. Intracellular parameters such as the replication and decay rates of HCV RNA, α, *r*, μ_*c*_ are the ones which the viral load is most sensitive to. The parameters that represents the export rate, ρ, and infected cell decay rate δ, are also important to define the viral load.

A baseline *in vivo* set of parameters was fixed based on the literature: α = 30 d^−1^, ρ = 8.18 d^−1^, δ = 0.14 d^−1^, and c = 22.3 d^−1^ were taken from Rong et al. (2013) and ϵ_*s*_ = 0.998 was taken from Guedj et al. (2013). The remaining parameters were estimated and their values are shown in Table 2.

**Table 2:** Model parameters estimated from fitting *in vivo* patient data.

**Param**.	**PAT 8**	**PAT 42**	**PAT 68**	**PAT 69**	**PAT 83**	**Mean**	**Range**	**Std**	**Conf**.
δ	0.58	0.64	0.1	0.47	0.62	0.48	0.1–0.8	0.199	0.209
μ_*t*_	0.89	0.89	0.88	0.89	0.89	0.89	0.8–1	0.004	0.004
*r*	1.49	1.1	5.08	2.24	1.61	2.3	1–6	1.435	1.506
μ_*c*_	2.55	1.72	3.38	3.15	2.39	2.6	1–6	0.587	0.616
ϵ_α_	0.928	0.909	0.992	0.936	0.924	0.937	0.9–0.99999	0.028	0.029
ϵ_*r*_	0.47	0.12	0.61	0.36	0.29	0.37	0–0.99999	0.165	0.173

*Mean values, range allowed for fitting, standard deviation and confidence interval (p = 0.05). We fixed α = 30 d^-1^ and κ_t_ = κ_c_ = 1. The parameters ϵ_α_ and ϵ_r_ are unitless, other parameters units, per day*.

**Figure 9:**
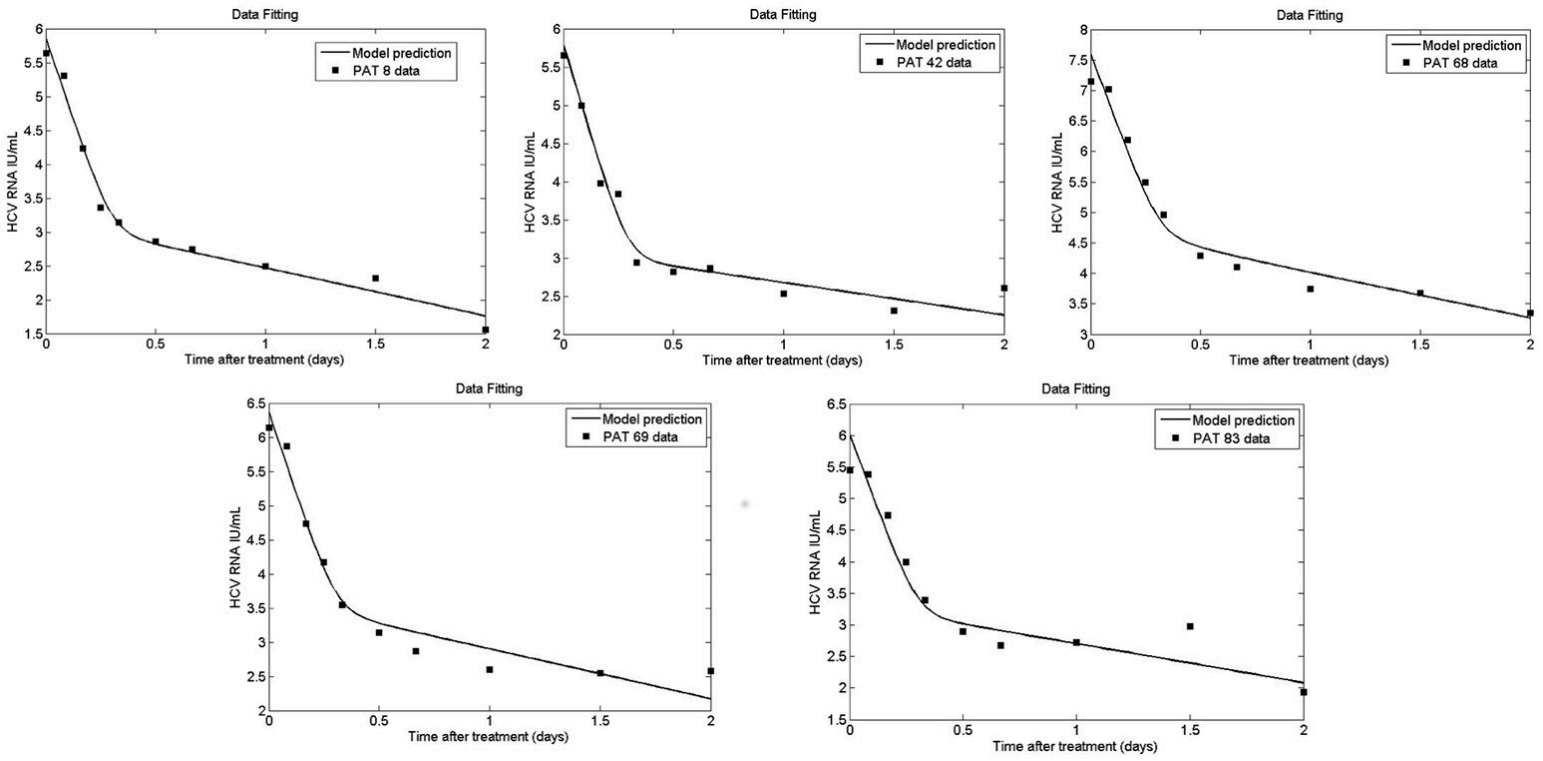
Fit of coupled multiscale model (solid line) to patient viral load data (squares) from Guedj et al. (2013). All 5 patients were treated with one dose of 10 or 100 mg of daclatasvir. The best-fit parameters are shown in Table 2.

**Figure 10:**
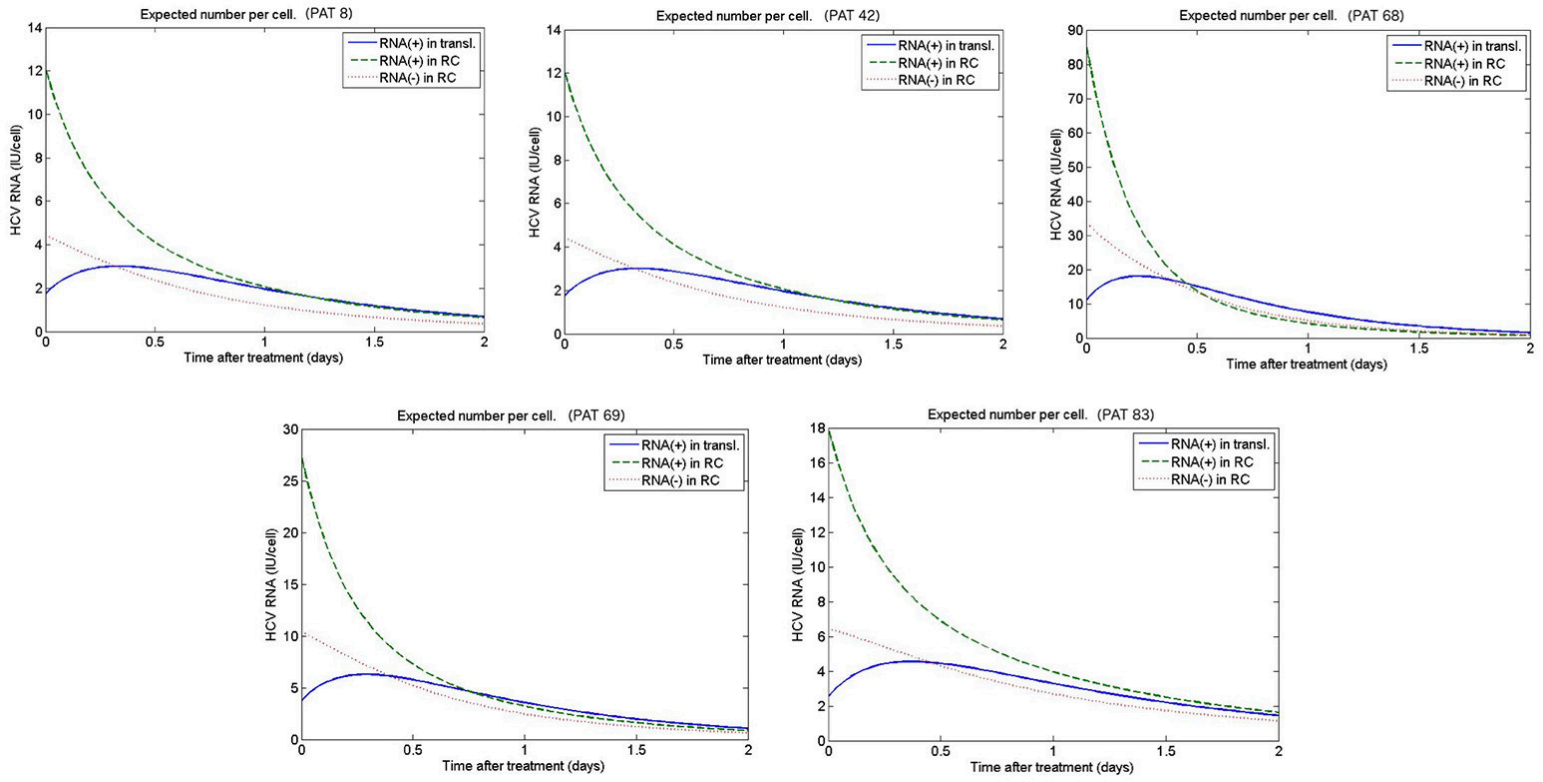
Predicted intracellular HCV RNA obtained from fitting the patient data from Guedj et al. (2013). The best-fit parameters are shown in Table 2.

Figures 9, 10 depict the results obtained with the multiscale model for each patient. We fixed the replication rate of positive strand HCV RNA α = 30 d^−1^ and considered no enhancement in HCV RNA decay with therapy, κ_*t*_ = κ_*c*_ = 1. Our model predicted that initiation of therapy affects the replication of both positive and negative strands and that initially there is a slightly increase in the number of positive strand HCV RNAs used for translation (Figure 10). This increase is most likely due to the fact that DCV effectively blocks secretion of positive strands thus allowing them to accumulate in the cytoplasm. Therapy also blocks the appearance of new replication complexes, which only decrease in the presence of the drug (Figure 10).

## Discussion

HCV infection and treatment has been modeled using variants of the basic model of viral infection starting with the work of Neumann et al. (1998). This initial ordinary differential equation model was followed by others and various clinical applications were shown (Layden et al., 2003; Layden-Almer et al., 2003; Powers et al., 2003; Ribeiro et al., 2003; Dixit and Perelson, 2004; Dahari et al., 2005, 2006, 2007a,b; Shudo et al., 2008a,b; Dahari et al., 2009; Reluga et al., 2009). These models were all based on the standard treatment at the time using type I interferon alone or in combination with ribavirin. When new small molecule inhibitors of HCV replication, such as the protease inhibitor telaprevir, were introduced parameters that were thought to reflect the host response to infection, such as the loss rate of infected cells (Guedj and Perelson, 2011) and the clearance rate of free virus (Adiwijaya et al., 2009) were found to change with the drug being used. To make sense of these findings a multiscale model was introduced by Guedj et al. (2013) that showed that the protease inhibitor telaprevir and the HCV NS5A inhibitor daclatasvir affected both viral replication and viral production. The Guedj et al. model only included postive strand HCV RNA and did not distinguish between the various functions of this RNA. However, the model showed that to fully understand the modes of action of anti-HCV drugs one would need to develop more detailed models of the viral lifecycle and couple them to models of cellular infection. Here we have done just that.

As negative-strand HCV RNA is only synthesized during viral replication, it should be considered a more reliable marker of viral replication than positive-strand HCV RNA (Yuki et al., 2005). In this work, the dynamics of negative-strand HCV RNA during replication was added to a multiscale age-structured model of HCV infection to better represent the steps of HCV replication inside of infected cells. Moreover, the addition of positive-strand RNA used for translation to the model is a new feature that allowed us to understand the initial decay in positive strand HCV RNA observed in *in vitro* experiments (Keum et al., 2012; Binder et al., 2013) before viral replication expanded the population of positive strands. This pool of HCV RNA is also a possible target of therapy and hence it is valuable to represent it in models. Another novel feature of our model was that we modeled the rate of export of positive strand HCV RNA not as a constant but rather as an increasing function of the time a cell has been infected. In this way, the initial positive strand RNA used to infect a cell has time to replicate before it is assembled into virions. The intracellular model was fit to two different *in vitro* experiments and was able to account for the intracellular dynamics seen in both as well as for the amount of positive strand HCV RNA secreted as virions into the medium in the experiment by Keum et al. (2012).

A sensitivity analysis of both the intracellular model and the multiscale model was performed indicating that the results are more sensitive to some parameters than others. In particular, the viral load is sensitive to the choice of intracellular parameters. The choice of parameters to be estimated or fixed was based on the availability of their values in the literature and which were more influential in determining the viral load during the sensitivity analysis.

The multiscale model presented here was able to reproduce the viral load during therapy and also the intracellular concentrations of positive and negative strands of HCV RNA observed during *in vitro* transfection experiments. Interestingly, the estimates of some parameters made from *in vitro* experiments were similar to estimates made from patient data. For example, we estimated that the replication rate constant for negative strand HCV RNA in the highly permissive Huh7-Lunet cells was 2.1 d^−1^, whereas our *in vivo* estimates varied between 1.1 d^−1^ and 5.1 d^−1^ with a mean of 2.3 d^−1^. Similarly, we estimated that the rate of decay of replication complexes, μ_*c*_ in Huh7-Lunet cells was 3.4 d^−1^, whereas our *in vivo* estimates ranged between 1.7 d^−1^ and 3.4 d^−1^, with a mean of 2.6 d^−1^. The estimate of the rate of decay of positive strands used for translation differed significantly between *in vivo* and *in vitro*, possibly due to more efficient depletion of positive strands *in vivo* by packaging into virions and secretion.

The model allows the effects of therapy to be estimated in terms of the targets: production of positive and negative stranded HCV RNA, secretion of new virions, and the enhancement in degradation of both strands of HCV RNA. Our estimate of the effectiveness of daclatasvir (DCV) treatment in blocking positive strand synthesis was between 0.91 and 0.99, whereas in Guedj et al. the mean was 0.99. More strikingly, we estimated that DCV was not nearly efficient in blocking negative strand synthesis, with estimates of ϵ_*r*_ ranging from 0.12 to 0.61 with a mean of 0.37. Thus, our model predicts that the NS5A inhibitor DCV is not very effective at blocking negative strand synthesis. This is consistent with the *in vitro* finding of McGivern et al. (2014) that NS5A inhibitors have no activity against preformed replication complexes and only inhibit the formation of new ones. If this is also true *in vivo*, then production of negative strand HCV RNA from existing replication complexes would continue in the presence of an NS5A inhibitor yielding a very low effectiveness of DCV in blocking this step of the HCV life cycle. However, preformed replication complexes also produce positive strands and why this production seems to be efficiently inhibited remains to be explained.

In summary, we have developed a new multiscale model of HCV replication and spread by cellular infection. The model is more realistic than the simple model developed by Guedj et al. (2013) that only contained positive strand RNA and more realistic than the prior model of Guedj and Neumann, which tracked positive strand RNA and replication complexes (Guedj and Neumann, 2010) but that was never fit to data. Here we showed that a model with positive strands used for translation separate from those used for replication as well as negative strands could fit both *in vitro* and *in vivo* data. More tests and refinement of the model may be needed, but it seems apparent that one does not need to introduce the complexity of the Binder model (Binder et al., 2013) or the earlier Dahari et al. model (Dahari et al., 2007c), both of which modeled HCV replication in enormous detail, in order to explain the *in vitro* and *on vivo* data analyzed here.

## Author contributions

AP, JC, and JG contributed to conception and design of the study; BQ worked on the simulations, performed the statistical analysis and wrote the first draft of the manuscript; JH developed the MATLAB® solver and wrote sections of the manuscript. All authors contributed to manuscript revision, read and approved the submitted version.

### Conflict of interest statement

The authors declare that the research was conducted in the absence of any commercial or financial relationships that could be construed as a potential conflict of interest.
